# Classification and quantification of full vs. empty adeno-associated virus reference capsids via infrared attenuated total reflection spectroscopy

**DOI:** 10.1007/s00216-026-06429-x

**Published:** 2026-03-24

**Authors:** Silke Lehner, Robert Stach, Robin Nilson, Stefan Kochanek, Astrid Kritzinger, Harald Sobek, Vjekoslav Kokoric, Boris Mizaikoff

**Affiliations:** 1 Institute of Analytical and Bioanalytical Chemistry, University of Ulm, Albert-Einstein-Allee 11, 89081 Ulm, Germany; 2 Hahn-Schickard, Sedanstraße 14, 89077 Ulm, Germany; 3 Department of Gene Therapy, University of Ulm, Helmholtzstraße 8/1, 89081 Ulm, Germany; 4 H. Sobek, Dinglingerstraße 10/3, 88400 Biberach, Germany

**Keywords:** Gene therapy, Adeno-associated virus, Infrared attenuated total reflection

## Abstract

Gene therapy is based on the introduction of nucleic acids into cells using vectors to enable therapeutic benefits. Hereby, adeno-associated virus (AAV) vectors are preferred for in vivo gene delivery to target cells due to their stability, their ability to transduce a broad range of cell types, and the long-term expression of vector-encoded therapeutic genes in slowly or non-replicating cells. However, large-scale production and quality control during and after manufacturing represent significant challenges. Currently, methods for the analysis of AAVs are rather complex, laborious, expensive, and poorly standardized. Most analyses have to be performed off-line and are not suitable for adaptation toward real-time monitoring during the manufacturing process. Optical analytical methods such as infrared attenuated total reflection (IR-ATR) spectroscopy offer a promising alternative serving as a rapid, cost-efficient, and non-destructive analytical technique with potential on-line monitoring capabilities. The present study fundamentally demonstrates the feasibility of IR-ATR spectroscopy to classify and quantify reference standard materials of full vs. empty AAV type 2 virus capsids. Chemometric methods enable rapid and automated processing of the obtained spectra using specifically partial least squares regression (PLS-R) models. Excellent classification results were achieved, unambiguously identifying samples with predominantly empty capsids vs. those containing predominantly full capsids. The obtained results confirm that IR-ATR spectroscopy in combination with multivariate data evaluation strategies is indeed capable of providing information on the presence of genome-containing virus particles along with the corresponding genomic titer. This analytical strategy may potentially translate into a real-world on-line bioreactor process analytical monitoring technology (PAT) augmenting viral vector production scenarios.

**Graphical abstract:**

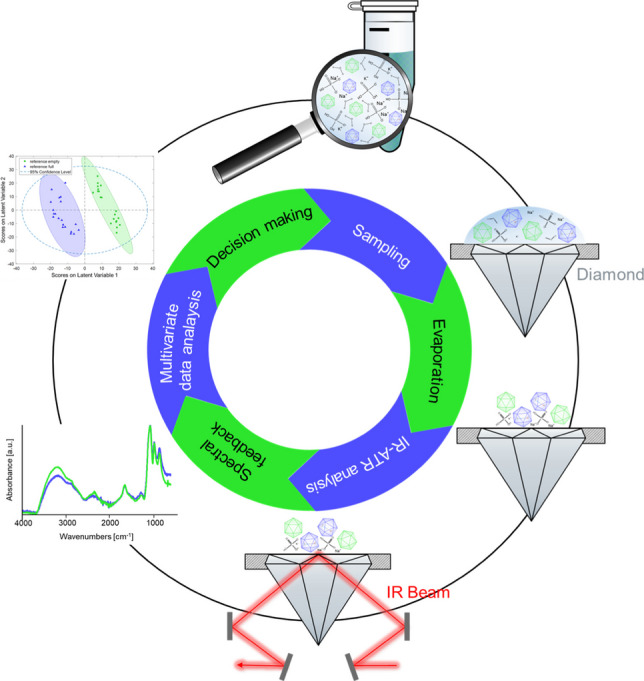

**Supplementary Information:**

The online version contains supplementary material available at 10.1007/s00216-026-06429-x.

## Introduction

According to Global Genes®, about 30 million people in the USA suffer from rare diseases. Approximately 80% of those diseases have a genetic cause and are inherited. Currently, for most of these diseases, there is no treatment available [1]. Gene therapy is based on the introduction of nucleic acids ex vivo or in vivo into cells by using vectors for gene delivery and expression, thereby enabling therapeutic benefit. In particular, for patients with monogenic inherited disorders—but in the future also with many other acquired diseases—gene therapy offers hope [2, 3].

Vectors used in gene therapy are grouped into non-viral and viral vectors. Viral vectors based on adeno-associated virus (AAV) are amongst the most widely used vectors in research, preclinical, and clinical development, offering several advantages over other vector types [4]. Besides the low pathogenicity in humans, their genome does not chromosomally integrate in transduced cells but persists as an episome, and long-term gene expression is achieved in cells undergoing no or little replication. In addition, the vector genome and capsid can easily be manipulated and modified, and they are characterized by an inherent physical stability [2, 4, 5].

In order to transfer AAVs to the clinic, scalable manufacturing routines considering good manufacturing practice (GMP) conditions are a prerequisite to achieve consistent quality of the therapeutic product. Consequently, accurate and standardizable analytical methods are mandatory for monitoring the manufacturing process, the products, and potential byproducts. Currently, most applied analytical methods are time-consuming, cost-intensive, require complex sample preparation, and offer only off-line measurements [6].

However, real-time monitoring of the AAV manufacturing process would be preferable to rapidly adapt process parameters if required. This could potentially be realized via infrared (IR) spectroscopy. This non-destructive analytical technique provides results rapidly and requires only minute amounts of samples with minimal sample preparation time. Due to the complexity of the matrix in bioprocesses, chemometric data analysis strategies need to augment spectroscopic measurements for obtaining multi-analyte information close to real time. Alternatives to classical chemometric methods, such as deep learning or spectroscopy machine learning (SpectraML), are already being used for the investigation of biological samples. These approaches aim to learn relevant spectral features directly from high-dimensional data and can be considered as complementary data analysis strategies, particularly for complex variable matrices [7–9]. Independent of the complexity of the data evaluation model, the data evaluation can be fully automated, rendering IR spectroscopy augmented by appropriate data evaluation routines an alternative method complementing currently used routine techniques, such as but not limited to quantitative polymerase chain reaction (qPCR), enzyme-linked immunosorbent assay (ELISA), or analytical ultracentrifugation (AUC) [10, 11].

A major bottleneck in AAV analytics is the lack of easy-to-implement methods for the determination of the ratio between genome-containing (“Full”) and genome-lacking (“Empty”) AAV particles, and—in a next step—to differentiate partially filled vs. overfilled capsids. During vector production, empty particles may represent up to 95% of the total particles. Empty particles do not provide a therapeutic purpose due to the lack of a genome coding for the therapeutic protein. In addition, administering large amounts of non-therapeutic AAV particles to a patient may lead to immunogenicity-linked adverse events. Consequently, it is of utmost importance to remove empty particles during product purification. The ability to monitor the entire biomanufacturing process and the final drug product according to the presence and amount of full and empty particles is an essential prerequisite [5, 12, 13].

Various methods for characterization of AAV capsids and genome content have been developed. The most commonly used methods include the combination of qPCR and ELISA [14] and uranyl acetate negative staining with subsequent analysis via transmission electron microscopy (TEM) [15, 16]. Another approach was described in 2003, when Sommer et al*.* succeeded in quantifying full and empty AAV capsids in solution using ultraviolet (UV) absorption spectroscopy [14]. In addition, Raman spectroscopy has demonstrated promising results as an in-line process analytical technology (PAT) tool for monitoring critical process parameters and for capsid titer determination during AAV upstream process [17]. More recently, the proportion of empty and genome-containing capsids has been determined using cryo-electron microscopy (cryo-EM). Visual identification and quantification were performed using 2D and 3D classification techniques [18]. Chromatographic separation of full and empty particles based on charge was found to be type-specific [19–21]. As previously mentioned, each of these methods has certain disadvantages, presents challenges for standardization, and lacks in potential as an on-line PAT. A current gold standard method is analytical ultracentrifugation (AUC), which takes advantage of the sedimentation velocity (SV) of full vs. empty capsids. Due to different buoyancy densities of the capsids, Burnham et al*.* were able to distinguish full, intermediate, and empty particles [22]. Charge detection mass spectrometry (CDMS) is an alternative method for this purpose [15]. However, both techniques have drawbacks, especially in quality control laboratories that must comply with GMP regulations, including relatively low throughput data acquisition, to date a lack in GMP compliance, and again limited utility as PAT. In 2021, McIntosh and co-workers demonstrated not only quantification, but also further characterization of AAV vectors using size exclusion chromatography coupled with multiangle light scattering (SEC-MALS) [23]. However, intermediate capsids could not be resolved. Last but not least, mass photometry was introduced by Wu et al*.* for the quantification of different filling states of AAVs. However, to date, the analysis procedure requires purified samples [13, 24].

Consequently, there is significant interest in developing methods that facilitate continuous monitoring and characterization of AAV particles either at-line (i.e., close to the sampling point), on-line (i.e., at the sampling point), or—ideally—in-line (i.e., directly integrated into the process). Continuous monitoring is a PAT strategy for consistently following the quality of the desired product in a bioreactor, enabling tuning of the production process in real time and for determining optimized harvesting times [6, 25]. In 2022, Schorer et al*.* published first promising results on the detection of viral material using IR spectroscopy without any additional sample preparation, albeit on protective face masks. Using multivariate data analysis, the classification of water, proteins, and viral particles was demonstrated without sample preparation or complex test equipment [26]. Complementarily, IR spectroscopy is also known as an excellent at-line, on-line, or in-line PAT method for real-time monitoring in quality control scenarios with applications in the food industry [27] or during the production of (bio)pharmaceuticals [28, 29].

The focus of the present study was the first demonstration of the feasibility and utility of infrared attenuated total reflection (IR-ATR) spectroscopy augmented by multivariate data analysis for the differentiation and quantification of full vs. empty AAV reference particles (AAV type 2) after solvent evaporation.

## Experimental section

### Materials and reagents

AAV2 standard reference materials with empty capsids (1.27 × 10^12^ VP per mL; 99.5% empty capsids) and full capsids (1.82 × 10^11^ GC per mL; 71.2% full capsids) were purchased from Vigene Bioscience (Rockville, USA). Both samples are present in a PBS storage solution. Buffer solution (potassium dihydrogen phosphate/disodium hydrogen phosphate, pH 7.00) was supplied by Merck KGaA (Darmstadt, Germany). All materials and reagents were used without any further purification and preparation. In the following, the buffer solution is abbreviated as PBS.

### Instrumentation

All measurements were performed using a portable FTIR spectrometer (ALPHA II Touch; Bruker Optics, Ettlingen, Germany) equipped with a deuterated L-alanine triglycine sulfate (DLaTGS) detector and a single-reflection ATR module (PLATINUM-ATR; Bruker Optics, Ettlingen, Germany). Absorbance spectra were recorded in the range of 4000 to 400 cm^−1^ with a spectral resolution of 2 cm^−1^ averaging 128 scans for each spectrum. Measurements were performed at room temperature versus air as the background. The spectrometer was operated by OPUS 8.5 software package (Bruker Optics, Ettlingen, Germany).

### Measurements

**Fig. 1:**
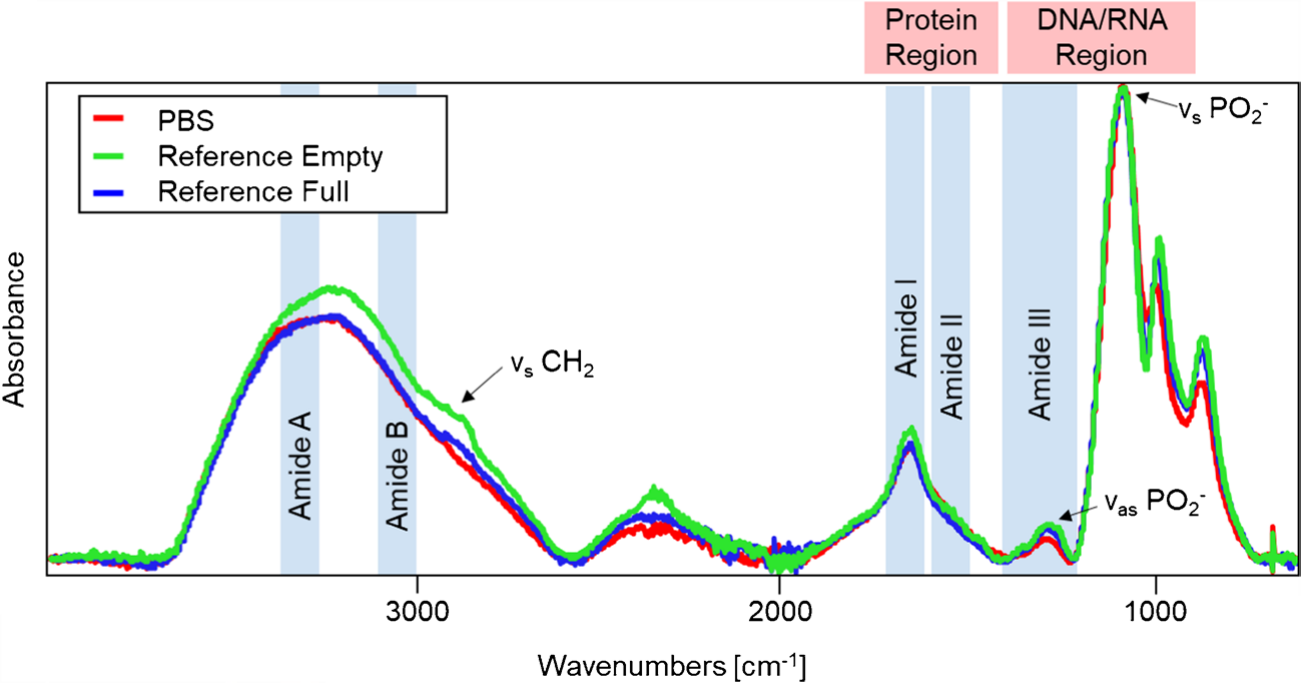
Exemplary IR-ATR spectra of the residues after water evaporation of PBS buffer (red), and AAV2 reference materials with empty (green) and full capsids (blue), along with the peak assignment. Atmospheric compensation, baseline correction, and normalization were applied to all spectra

5 µL of the sample was pipetted on the single-bounce diamond (area of 2 × 2 mm) of the ATR element. No further sample preparation was necessary. At least eight spectra were recorded. To obtain technical replicates, three repeat measurements were performed for each sample. Quantification was performed using calibration and validation data. For calibration, a dataset of ten mixtures (mixtures A–J) of PBS and AAV2 reference materials with empty and full capsids was prepared. Validation was performed using five mixtures (mixtures K–O). The exact volumes and the titers are shown in Table S1 (see SI). After each measurement, the ATR crystal was cleaned using demineralized water and isopropyl alcohol. It is important to note that all IR-ATR spectra were recorded after evaporation of water at the dry residues remaining at the ATR waveguide surface. IR-ATR spectra of evaporated PBS (red), AAV2 empty (green), and full (blue) capsid reference materials are shown in Fig. 1, emphasizing the need for multivariate data analysis given the only subtle spectral differences.

### Data evaluation and pre-processing

Data acquisition was followed by pre-processing of the complex IR spectra recorded to reduce or remove unwanted interference, resulting in optimal model performance. This was performed using Essential FTIR (OPERANT LLC, Madison, USA), OPUS, and MATLAB (Release 2021, MathWorks, Natick, USA) with PLS toolbox 8.8.1 (Eigenvector Research Inc., Manson, USA). For the evaluation of the data, the first three recorded spectra of each data set were neglected, as the samples were not dried completely. After 12 min (at normal conditions described above), no more water bands are evident, confirming a dry residue at the ATR crystal surface. Therefore, the resulting IR spectra correspond to a mixture of the salts initially contained within the PBS buffer matrix and dried virus particles. In the next step, atmospheric compensation was applied to all spectra using OPUS to remove the spectral features of water vapor (2000–1300 cm^−1^). This is necessary as the fine absorption lines coincide with the amide I–III region of proteins (see Table 1) [30]. All processed spectra were limited to the fingerprint region in the spectral range of 1800–800 cm^−1^ to avoid evaluating spectral segments containing no or little molecularly relevant signatures/information. Besides, the predictive power of the established model will be improved by excluding irrelevant spectral regions. As shown in Fig. 1, this spectral range covers the protein and DNA/RNA region. Here, three characteristic amide bands can be found, namely amide I (1700–1600 cm^−1^), amide II (1600–1500 cm^−1^), and amide III (1400–1200 cm^−1^). Furthermore, the asymmetric (~1225 cm^−1^) and symmetric (~1080 cm^−1^) phosphate stretching vibrations are located here [11, 31, 32]. In addition to the expected bands of proteins and DNA, there are also other P-O-H vibrations in this region, caused by the salts present in PBS [33, 34]. This explains why pure PBS has bands in this spectral range. The number of data pre-processing steps was kept at a minimum to avoid overfitting while ensuring optimal model performance. For classification and quantification, two different pre-processing workflows were applied. For classification, the pre-processing comprised baseline correction, normalization, smoothing, and autoscaling. Baseline correction, namely Weighted Least Squares automatic baseline removal (AWLS, order = 3), was implemented first to adjust the baseline. Subsequently, the spectra were normalized using 1-Norm normalization (area = 1). Spectral smoothing was then applied using a Savitzky-Golay filter. As a last step, autoscaling was performed, which involves mean centering followed by dividing each variable to unit standard deviation [35].

For quantification of the reference materials, a manual baseline correction was applied instead of AWLS. As a next step, the data was normalized (1-Norm, Area = 1) to improve the signal-to-noise ratio. Next, Savitzky-Golay filtering was applied for de-noising. By varying the order of the filter to two, Savitzky-Golay differentiation was used to resolve overlapping bands, which facilitates impulse searching [36]. This step was necessary to emphasize subtle spectral differences. As a last pre-processing step and prerequisite for PLS-R, Autoscale was used.

**Fig. 2:**
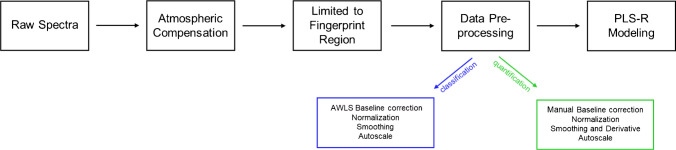
Pre-processing workflow of spectral data prior to PLS-R modeling

All preprocessed data were subsequently evaluated using PLS-R. The complete data pre-processing workflows are schematically illustrated in Fig. 2.

**Table 1:** Normal modes of the amide groups [31, 32]

Wavenumber $\widetilde{\upnu }$ (cm^−1^)	Assignment
3310–3270	Amide A
3100–3030	Amide B
1700–1600	Amide I
1600–1500	Amide II
1400–1200	Amide III

## Results and discussion

As mentioned above, previous studies by Schorer et al*. *[26] demonstrated that IR spectroscopy combined with multivariate data analysis can be used for the classification of viral materials. Evidence was provided that two different types of viral particles—AAV2 and virus-like particles (VLPs) derived from SARS-CoV-2—could be classified. The present study is focused on the differentiation between empty (i.e., DNA-lacking) and full (i.e., DNA-containing) viral particles of AAV2 using reference materials. IR-ATR spectroscopy was used to discriminate samples containing empty capsids vs. samples with full ones. Besides the presence or absence of the genome, also other sample differences had to be considered for quantification. On the one hand, the samples were different in their titer, i.e., the concentration of the contained viral particles. Reference materials with empty capsids are described by the number of viral particles (VP), whereas the reference materials with full capsids are defined by the number of genome copies (GC). Furthermore, neither sample contains 100% empty or full capsids. While the “empty” reference materials contain 99.5% empty capsids and 0.5% full capsids, the “full” reference material comprises only 71.2% filled capsids with a remaining fraction of 28.8% empty capsids.

### Classification of AAV2 empty vs. full capsids vs. PBS

First, IR-ATR spectra of AAV2 standard reference materials of empty and full capsids were analyzed (sample B and C in Supporting Information). The obtained spectra are shown in Fig. 2. As expected, both samples show characteristic—albeit only minutely different—spectral features in the wavelength regions relevant to protein and/or DNA absorption features. The main difference between empty and full AAV2 capsids should be the presence or absence of DNA. DNA is composed of a sugar-phosphate backbone and four different nitrogen bases. These biological constituents are evident in the fingerprint regime of the infrared spectra (see Table 2).

**Table 2:** Major infrared signals of DNA [11]

Wavenumber $\widetilde{\upnu }$ (cm^−1^)	Assignment
1660–1655	DNA C=O stretching; N–H bending
1610	C=C imidazole ring stretching
1578	C=N imidazole ring stretching
1230	DNA PO_2_^−^ asymmetric stretching
1089	DNA PO_2_^−^ symmetric stretching
1060, 1050	Ribose C-O stretching
1015	DNA ribose C-O stretching
970, 916	DNA ribose–phosphate skeletal motions

Low-concentrated virus particles present in a PBS buffer solution apparently result in only subtle spectral differences between empty and full capsids. Furthermore, some spectral signatures overlap within the characteristic regions for the amide I–III features. As visual discrimination is evidently not possible, chemometric methods such as PLS-R are inevitably needed for establishing reliable and robust classification models.

**Fig. 3:**
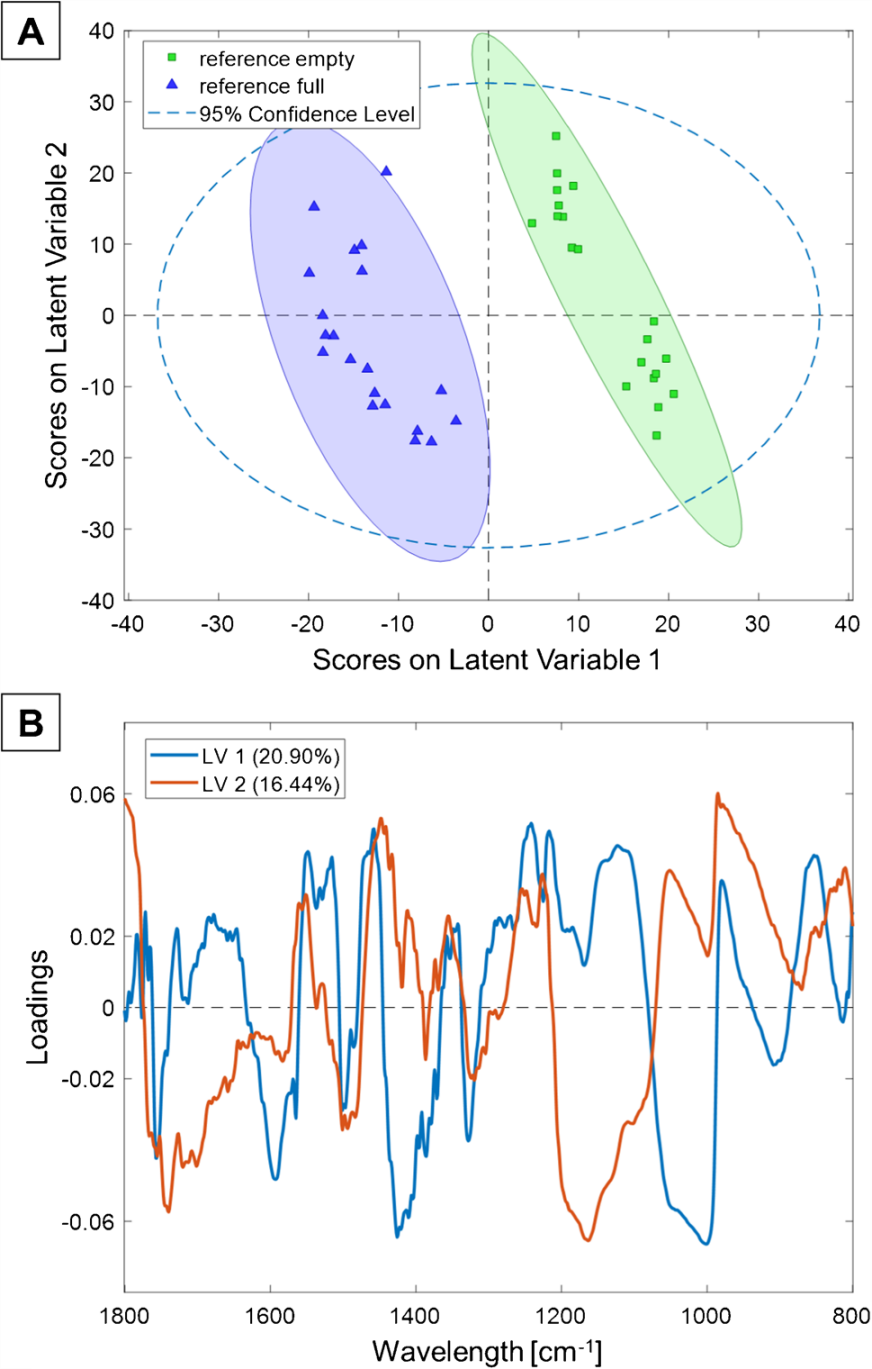
**A** Scores plot of PLS-R evaluation of Vigene AAV reference materials with empty AAV2 particles (green) vs. full (blue) AAV2 particles. The samples are unambiguously separated using LV 1 (20.9%) and LV 2 (16.4%). **B** Loadings vs. wavelength of LV 1 (blue) and LV 2 (red). (Pre-processing: Baseline correction, Normalization, Smoothing, and Autoscaling)

The PLS-R model shown in Fig. 3 is based on five latent variables (LVs) covering 83.73% of the total variance. The optimal number of LVs was selected by analyzing the root-mean-square error of calibration (RMSEC) and the root-mean-square error of cross-validation (RMSECV) vs. the number of LVs. The final model was chosen at the point where the RMSECV reached a minimum, and further increases in the number of LVs did not result in a significant reduction of the prediction error, thereby preventing the modeling of noise and overfitting. Using five LVs, the predictive performance of the established model could be maximized, and interferences could be suppressed. The obtained RMSEC and RMSECV for empty and full capsid samples, along with further classification results, are summarized in Table 3. While the RMSEC value indicates how well the model fits the calibration data, the RMSECV value provides information on how well the established model predicts unknown samples [37]. A value of almost 1 is obtained for the coefficient of determination (R^2^) for calibration and cross-validation, verifying a high predictive accuracy of the established model. Cross-validation (CV) was performed involving 10 splits using the venetian blinds method with a blind magnitude of 1.

As shown in Fig. 3A, the scores plot compares the scores of LV 1 and LV 2. AAV2 empty (green ellipsoid) and full (blue ellipsoid) particles with an unambiguous discrimination on LV1 are indicated by the distinct separation of their 95% confidence ellipses. Thus, the generated PLS-R model clearly responds to the minute differences in the obtained IR spectra. The absence or presence of genomic material, while not immediately evident by visual inspection, sufficiently affects the spectral properties, allowing for a reliable classification. The loadings on LV 1 and LV 2 are in good agreement with the expected spectral signatures for the AAV samples (see Fig. 3B).

A viral capsid is composed of a variety of structural proteins, which have their characteristic spectral signatures in the range of 1800–1400 cm^−1^. For full AAVs, a genome is present, which provides additional spectral signatures in the regime of 1400–900 cm^−1^. Evaluating the loadings in more detail, two pronounced signals are apparent. The peak at 1000 cm^−1^ can be attributed to the C-O stretching vibration of DNA ribose, which indicates full capsids containing a genome. Another distinct peak is located at approx. 1160 cm^−1^. In this wavelength range, the IR bands of amino acid side chains are located. These biological structures are components of proteins, which form the capsomers contained in the capsid of AAV [31]. Based on the fact that 5 LVs cover 83.73% of the total variance, it can be confirmed that unique spectral differences for full vs. empty AAV capsids can be extracted via IR-ATR spectroscopy.

**Fig. 4:**
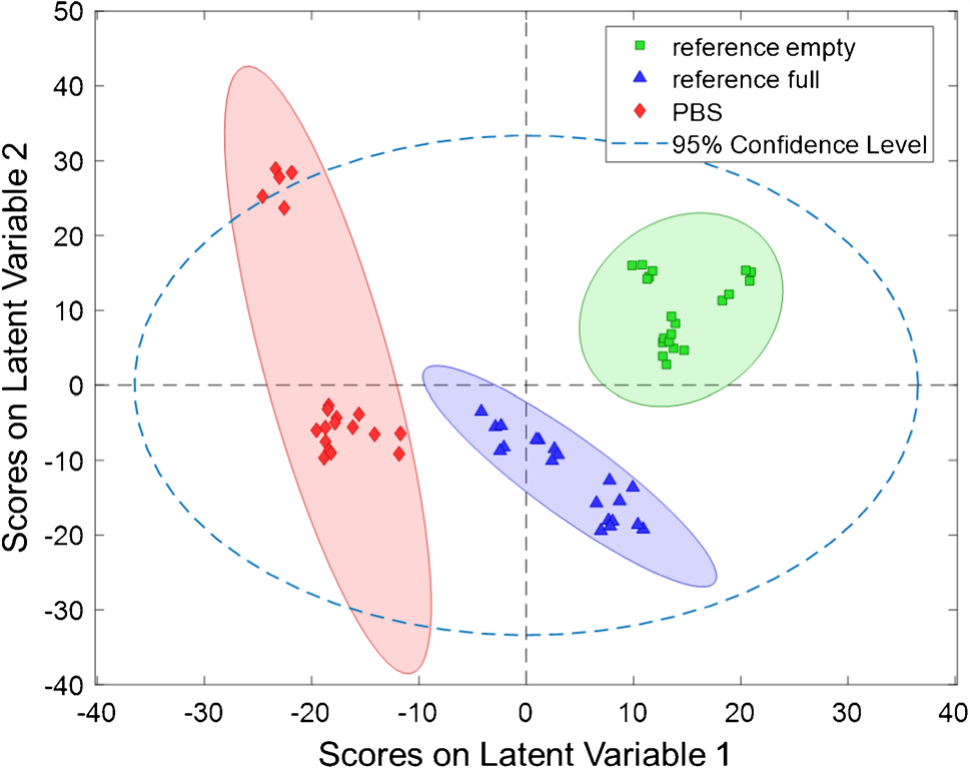
Scores plot of PLS-R evaluation of Vigene AAV reference materials with empty AAV2 particles (green) vs. full (blue) AAV2 particles vs. PBS residues (red). The classes show an excellent separation using LV 1 (21.4%) and LV 2 (17.8%). (Pre-processing: Baseline correction, Normalization, Smoothing, 2nd Derivative, and Autoscaling)

In a next step, PBS signatures were added to the model (Fig. 4; sample A in Supporting Information). Again, five LVs were used, resulting in a cumulative captured variance of 61.18%. Again, the 95% confidence ellipses do not overlap, indicating an unambiguous classification of the three analytes, i.e., full capsids vs. empty capsids vs. pure PBS. The scores plot demonstrates that the spectral signatures of PBS do not influence the capsid analysis and that interfering matrix effects can be excluded. PBS is separated from the viral particles on LV 1. RMSEC values ranging from 5.7% to 7.2% and RMSECV values within 7.9% and 11.7% were achieved. For R^2^, as expected, slightly reduced values were obtained vs. the first model, yet still with superior predictive accuracy (Table 3).

**Table 3:** Analytical figures-of-merit of the established PLS-R classification models based on IR-ATR spectra

Analyte	RMSEC (%)	RMSECV (%)	*R*^2^ Cal	*R*^2^ CV
Reference empty	3.46	5.43	0.99	0.99
Reference full	3.46	5.43	0.99	0.99
Reference empty	5.67	7.86	0.98	0.97
Reference full	7.16	11.68	0.95	0.89
PBS	6.77	9.47	0.98	0.96

The obtained results show an unambiguous discrimination based on the IR-ATR spectra of AAV2 empty capsids vs. AAV2 full capsids vs. PBS residues via a robust PLS-R model.

### Quantification of AAV2 full vs. empty capsids

**Fig. 5:**
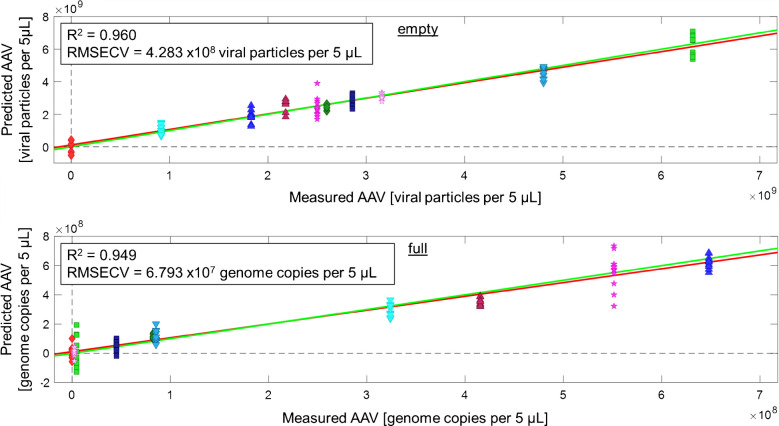
Multivariate quantitative calibration of ten sample groups containing PBS and AAV2 reference materials with empty and full particles. (Pre-processing: Baseline correction, Normalization, Smoothing, 1 st Derivative, Autoscaling)

Beyond discriminating full and empty capsids, it is even more relevant how many full capsids are present within a sample. Based on the classification results, another PLS-R model was trained for estimating the titer of empty and full capsids within minute 5 µL droplet samples. In contrast to the qualification model, a Savitsky-Golay filter was necessary to better resolve overlapping spectral features. The previously used data set (PBS, AAV2 empty and full) was used as a basis and extended using five additional sample mixtures of full vs. empty AAV capsids prepared (samples D–J in Supporting Information). The resulting RLS-R model is shown in Fig. 5.

Nine LVs were selected following the optimum RMSECV and RMSEP values. In total, the selected LVs cover 95.91% of total variance within the calibration dataset. Both obtained calibration functions confirm an excellent linear correlation between the experimentally measured and the predicted quantitative fraction of full vs. empty capsids in the samples, indicated by R^2^ values of 0.960 (empty particles) and 0.949 (full particles), respectively. Furthermore, an RMSECV value of 4.3 × 10^8^ viral counts for reference materials containing empty particles and an RMSECV of 6.8 × 10^7^ viral counts for reference materials containing full particles were obtained, which further highlights the quality of this quantitative model.

The results obtained confirm the predictive ability of the established model. In particular, samples C and E show that, based on the developed quantification model, even the presence of only a very small percentage of full capsids can be clearly determined, which underlines the accuracy of the developed measurement technique and the associated data evaluation model. Consequently, it is confirmed that only minimal differences in the IR spectrum caused by different concentrations of empty vs. full capsids—and consequently, also the proportion of the genome—using IR-ATR spectroscopy is indeed feasible. These findings for the first time establish the potential of IR-ATR spectroscopy as a real-time monitoring tool for quality control and as a PAT strategy in bioprocess monitoring, and in particular for the production of gene therapeutics. From a pharmacological point of view, the reliable quantification of full capsids is even more relevant, as for the administration of AAVs in gene therapeutics, only genome-containing particles should ideally be used, as empty particles may have adverse clinical effects. Therefore, it is essential to accurately and precisely verify the fraction of AAV particles that contain a genome during the (bio)production, and especially as a quality control criterium of the final gene therapy product [12].

**Fig. 6:**
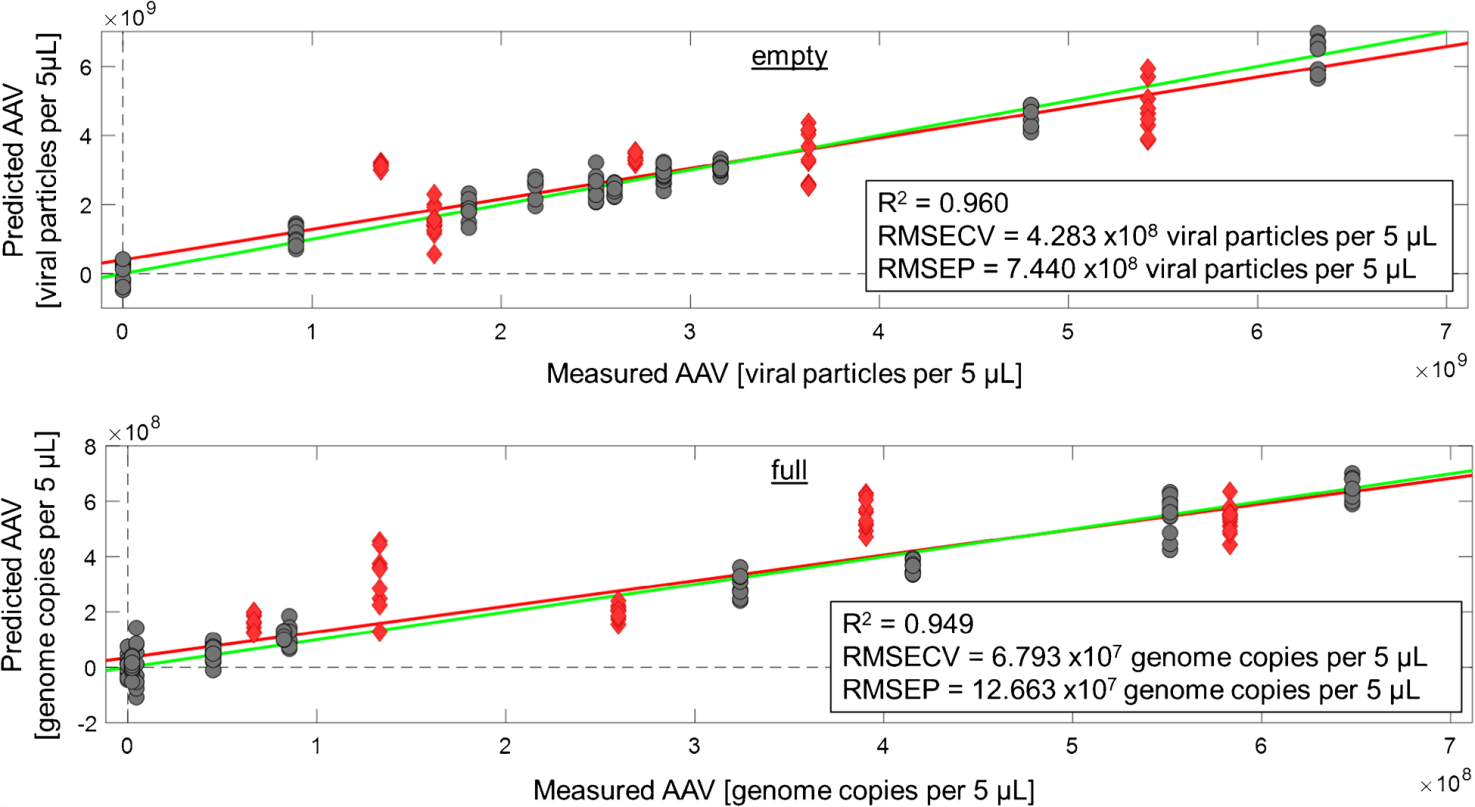
Multivariate validation of ten sample groups containing PBS and AAV2 reference materials with empty and full particles used for establishing the calibration model (grey dots) and five test mixtures only used for validation (red diamonds). (Pre-processing: Baseline correction, Normalization, Smoothing, 1 st Derivative, Autoscaling)

Finally, for validation, 5 mixtures (samples K–O in Supporting Information) were evaluated using the same model shown in Fig. 5. The prediction is shown in Fig. 6. The calibration data is indicated by grey dots and the validation data by red diamonds. RMSEP and RMSECV result in excellent values, i.e., 7.4 × 10^8^ viral counts for reference materials with empty capsids and 12.7 × 10^7^ viral counts for reference materials with full capsids.

## Conclusions and outlook

In this study, an innovative IR spectroscopic approach for classifying and quantifying viral capsids and the embedded genome was developed using infrared attenuated total reflection (IR-ATR) spectroscopy combined with appropriate multivariate data analysis routines (i.e., partial least squares regression; PLS-R). It was demonstrated that this analytical strategy provides robust and reliable models for the classification of full vs. empty AAV particles. Despite only minute spectral differences, an unambiguous and precise discrimination between genome-containing viral particles and empty capsids that do not contain DNA is facilitated. Additionally, a model for quantifying the absolute amount of full vs. empty AAV particles was established and tested using validation data from five independent sample mixtures. Next to the quantification of empty vs. full capsids, it was shown that even exceedingly low amounts of full viral capsids may be accurately characterized. Based on the inherent molecular selectivity and the unique spectral fingerprints in the mid-infrared spectral range, the simultaneous classification and quantification of full vs. empty AAV capsids was shown. In addition, it should be noted that only minimal sample preparation was necessary (10 s) along with short measurement times (5 min) for obtaining the demonstrated sensitivity, accuracy, and precision. Moreover, IR-ATR spectroscopy by its technical nature offers the benefits of a non-destructive measurement in a possibly compact/portable format, cost-effectiveness due to minimum maintenance, and the requirement of only minimal sample volumes (here, 5 µL). The obtained results represent a proof of principle for IR-ATR spectroscopy as a PAT strategy in large-scale (bio)processing, e.g., for future on-line monitoring during the biotechnological production of gene therapeutics and other biomedically relevant active pharmaceutical ingredients (APIs).

In summary, IR-ATR spectroscopy combined with multivariate data analysis strategies provides a promising first step toward automated quality control in biotechnological production scenarios, demonstrating the fundamental feasibility of distinguishing empty and full capsids under controlled conditions while avoiding complex sampling and measurement routines. However, the presented study is limited to purified samples and does not yet address the complexity of real bioprocess matrices. In a next development step, the established concepts will be further developed and validated, for at-line, on-line, and in-line monitoring scenarios during relevant manufacturing processes in biopharmaceutical industries. In addition, the measurements and predictive models shown herein have proven their capabilities in PBS buffer and will be tested and refined in a next step for analyses in more complex real-world matrices encountered during AAV manufacture. It is anticipated that augmenting the established data evaluation routines with deep learning algorithms or MSL addressing a wide range of bioprocessing matrices will facilitate adapting the developed fundamental routines to a variety of biotechnical production scenarios as a future routine PAT tool for the quality control of biopharmaceuticals.

## Supplementary Information

Below is the link to the electronic supplementary material.

Supplementary file1 (DOCX 18.7 KB)

## Data Availability

Available from the authors by request.
