# Supplementary material for: Classification and quantification of full vs. empty adeno-associated virus reference capsids via infrared attenuated total reflection spectroscopy
Source: Anal Bioanal Chem. 2026 Mar 24;418(11):3271–80. doi: 10.1007/s00216-026-06429-x (PMC13197263; doi:10.1007/s00216-026-06429-x)
Supplement: Supplementary file 1 — Supplementary file1 (DOCX 18.7 KB) [file 216_2026_6429_MOESM1_ESM.docx]

**Supporting Information: Classification and quantification of full vs. empty adeno-associated virus reference capsids via infrared attenuated total reflection spectroscopy**

Silke Lehner^1,2^, Robert Stach^2^, Robin Nilson^3^, Stefan Kochanek^3^, Astrid Kritzinger^3^, Harald Sobek^4^, Vjekoslav Kokoric^2^, Boris Mizaikoff^1,2,*^

^1^Institute of Analytical and Bioanalytical Chemistry, University of Ulm, Albert-Einstein-Allee 11, 89081 Ulm, Germany.
^2^Hahn-Schickard, Sedanstraße 14, 89077 Ulm, Germany.
^3^Department of Gene Therapy, University of Ulm, Helmholtzstraße 8/1, 89081 Ulm, Germany
^4^ H. Sobek, Dinglingerstraße 10/3, 88400 Biberach, Germany
*Corresponding author: boris.mizaikoff@uni-ulm.de

Table 1: Information about volume and titer of calibration (A - J) and validation (K - O) mixtures

|  | Volume in 15 µL sample | | | Titer in droplet | |
| --- | --- | --- | --- | --- | --- |
|  | *PBS [µL]* | *empty [µL]* | *full [µL]* | *empty  [VP ∙ 5 µL^-1^]* | *full  [GC ∙ 5 µL^-1^]* |
| A | 15 | 0 | 0 | 0 | 0 |
| B | 0 | 0 | 15 | 1.83 ∙ 10^9^ | 6.48 ∙ 10^8^ |
| C | 0 | 15 | 0 | 6.32 ∙ 10^9^ | 4.55 ∙ 10^6^ |
| D | 7.5 | 0 | 7.5 | 9.14 ∙ 10^8^ | 3.24 ∙ 10^8^ |
| E | 7.5 | 7.5 | 0 | 3.16 ∙ 10^9^ | 2.28 ∙ 10^6^ |
| F | 7.5 | 5.63 | 1.87 | 2.60 ∙ 10^9^ | 8.25 ∙ 10^7^ |
| G | 7.5 | 6.5 | 1 | 2.86 ∙ 10^9^ | 4.52 ∙ 10^7^ |
| H | 3 | 2.4 | 9.6 | 2.18 ∙ 10^9^ | 4.15 ∙ 10^8^ |
| I | 2.25 | 10.85 | 1.9 | 4.80 ∙ 10^9^ | 8.54 ∙ 10^7^ |
| J | 0 | 2.25 | 12.75 | 2.50 ∙ 10^9^ | 5.51 ∙ 10^8^ |
| K | 0 | 12 | 3 | 5.42 ∙ 10^9^ | 1.33 ∙ 10^8^ |
| L | 7.5 | 6 | 1.5 | 2.71 ∙ 10^9^ | 6.66 ∙ 10^7^ |
| M | 0 | 6 | 9 | 3.62 ∙ 10^9^ | 3.91 ∙ 10^8^ |
| N | 7.5 | 1.5 | 6 | 1.36 ∙ 10^9^ | 2.60 ∙ 10^8^ |
| O | 1.5 | 0 | 13.5 | 1.65 ∙ 10^9^ | 5.83 ∙ 10^8^ |
